# Nurse-led web-based self-management program to improve patient activation and health outcomes in patients with chronic low back pain: an acceptability and feasibility pilot study

**DOI:** 10.1186/s12912-024-02155-w

**Published:** 2024-07-31

**Authors:** Richard L. Skolasky, Sarah Nolan, Raven Pierre, Paige Vinch, Janiece L. Taylor

**Affiliations:** 1 Department of Orthopaedic Surgery, Johns Hopkins University School of Medicine, Baltimore, MD USA; 2 Johns Hopkins University School of Nursing, Baltimore, MD USA

**Keywords:** Chronic low back pain, Disability, Patient activation, Physical function, Self-management

## Abstract

**Background:**

Patients with chronic low back (cLBP) pain report reduced physical function and ability to participate in social roles and are more likely to use opioid pain medications. While self-management interventions have been shown to support these patients, their effectiveness has been limited due to poor patient engagement. “Patient activation” encompasses the skills, knowledge, and motivation that a person has to manage their health. Supporting patient activation may improve the effectiveness of self-management for cLBP.

**Methods:**

In this single-masked pilot study of adults with cLBP, patients were randomized to receive either no intervention (control) or 6 weekly sessions of an evidence-based web-based self-management program (SMP) with or without health behavior change counseling (HBCC) using motivational interviewing. Participants were assessed at baseline and at 12 and 26 weeks using the Patient Activation Measure, Oswestry Disability Index and PROMIS physical function, social role participation, and pain interference. We assessed acceptability and feasibility based on recruitment, session attendance, and follow-up.

**Results:**

Of 187 individuals screened, 105 were eligible and 34 were randomized to control (*n* = 12), SMP (*n* = 4), or SMP + HBCC (*n* = 18). The population had 19 women, 22 patients married or living with significant other, 13 Black or African American patients, and 4 Hispanic or Latino patients. Participants had a mean (SD) Oswestry Disability Index score of 42 (12), moderate impairments in physical function (40 (6.6)) and social roles (45 (10)), and moderately severe pain interference (61 (6.7)). Of 22 participants receiving SMP sessions, 20 participated in at least 1, 15 participated in at least 3, and 7 participated in all 6 sessions. Loss to follow-up was 6 over the 26-week study. Participants in the SMP and SMP + HBCC groups had at least medium effect size improvements in Patient Activation Measures and small-to-medium effect size improvements in Oswestry Disability Index scores and physical function and large effect size improvement in social roles at 12 weeks. Improvements persisted in the SMP + HBCC group at 26 weeks.

**Conclusions:**

A web-based SMP is acceptable and feasible in this population. Participants who received augmentation with HBCC had persistent improvements in health outcomes at 26 weeks.

**Trial Registration:**

ClinicalTrials.gov Identifier NCT06236529 (2/1/2024 – retrospectively registered).

**Level of Evidence:**

3.

**Supplementary Information:**

The online version contains supplementary material available at 10.1186/s12912-024-02155-w.

## Background

In the United States, approximately 80% of adults experience at least 1 episode of low back pain (LBP) during their lifetime [1]. LBP accounts for roughly 5% of all health care visits [2, 3] and an estimated $135 billion in spending, exceeding diabetes, heart disease, and Alzheimer’s disease [4]. Despite intensive clinical efforts, the prevalence of chronic LBP (cLBP) is increasing at a faster rate than that of most other health conditions, and as the population ages, it will likely accelerate [5]. LBP is the most common diagnosis for which opioids are prescribed [6, 7], despite a lack of evidence of long-term benefit [7, 8]. Between 2000 and 2010, visits for opioid prescriptions for noncancer pain nearly doubled to 20% of all health care visits [8], despite clinical care guidelines highlighting the need for non-opioid and nonpharmacologic treatment as the first-line treatment for these patients [9]. Similar patterns in visits for opioid prescriptions have been observed in patients with LBP [7].

Self-management interventions can be effective in managing symptoms, improving health outcomes, and supporting health promotion behaviors and decisions among people living with chronic pain conditions [10–13]. Self-management is defined as tasks or strategies within the living environment that promote health through 5 core activities: problem-solving, decision-making, resource utilization, partnerships with health care providers, and taking action [11–14]. Researchers have found that patients with cLBP typically request a discussion of self-management and the disease process with their clinicians [15]. Although self-management interventions led by nurses [16–18] or other health professionals [19] or delivered via the Internet [20] have been shown to improve pain intensity among people with cLBP, evidence of the impact of these interventions on pain-related disability (i.e., the ability to perform routine tasks) is limited. Self-management programs (SMPs) tailored to people with cLBP have the potential to improve outcomes in pain-related disability, particularly when SMPs support patient engagement, but it is not known which SMPs are most beneficial for adults with cLBP or which components of SMPs are necessary for these programs to be effective.

Patient activation is a crucial component of self-management that directly affects engagement, utilization, and the success of self-management interventions. This emergent construct reflects patients’ sense of control over their health and health care, their understanding of treatment alternatives, and their confidence and motivation to take action. Patients with high activation are able to set personal goals, identify challenges, and create a comprehensive action plan to meet their goals [21]. People who are less activated have fewer self-management skills and are more likely to experience health declines [22]. Self-management interventions without activated participants often result in a transfer of knowledge but not an improvement in self-management skills and health behaviors [23].

Research has shown that patient activation can be increased in chronically ill patients [24, 25] and those undergoing surgery for back-related conditions [26, 27]. Although there are studies that demonstrate the effectiveness of interventions that focus on activation in people with cLBP, however, they tend to be observational [28, 29]. Work is needed to test the effectiveness of structured interventions that target patient activation in patients with cLBP because such interventions could improve self-management and outcomes [23].

Our study aimed to fill this important gap. We tested an intervention that incorporates evidence-based strategies to improve patient activation in an effort to tailor self-management strategies to people living with cLBP and to determine the potential for a larger clinical trial in this population. We designed a nurse-led, evidence-based self-management program, augmented with health behavior change counseling (HBCC) and delivered via synchronous, video-enabled, web-based platform. We examined the acceptability and feasibility of implementing this program in this patient population and the participants’ perceived barriers to and facilitators of using self-management. We believe this study has the potential to lead to long-term improvements in self-management outcomes that are often not achievable in the absence of patient activation.

### Theoretical framework

We used Andersen’s behavioral model [30], the schematic representation of self [31], and the concept of patient activation [32] to guide the study. The ability to use self-management is related to a person’s predisposition toward self-management strategies, barriers to and facilitators of self-management, personal characteristics (resources, health status), and access to health care and self-management strategies. Patient activation must be in place in order for the key attributes of self-management to be fulfilled. The development and use of evidenced-based interventions to increase patient activation is essential to ensure that participants are engaged and that their individual circumstances are accounted for in the development and implementation of self-management strategies. We conducted a self-management intervention that included group-based education and exercise classes. Health behavior change was used to improve patient activation. Improvements in patient activation may improve participation in self-management, potentially leading to improvements in pain and quality of life [12, 33, 34].

## Methods

### Participants

This pilot study was a single-masked randomized controlled trial of adults with cLBP conducted at an academic medical center from July 2022 through April 2023. Institutional review board approval was received (Johns Hopkins Medicine Institutional Review Boards, Protocol # IRB00242529), and patient informed consent was provided before participation.

Patients were adults (≥ 18 years of age) seen in a specialty or primary care practice. They had a diagnosis consistent with nonspecific low back pain and, upon screening, endorsed cLBP based on 2 questions in the National Institutes of Health Task Force on Research Standards for Chronic Low Back Pain [35] questionnaire: (1) “How long has low back pain been an ongoing problem for you?” and (2) “How often has low back pain been an ongoing problem for you over the past 6 months?” Responses of “greater than 3 months” to question 1 and “at least half the days in the past 6 months” to question 2 were required. Patients were included if, at the time of enrollment, they (1) were ≥ 18 years old, (2) had chronic LBP, (3) made at least 1 outpatient visit in the preceding 90 days, (4) made a provider visit in an outpatient/ emergency setting, (5) experienced worst back pain of ≥ 4/10 points, (6) had an Oswestry Disability Index score ≥ 24%, and (7) could speak English. Patients were excluded if, at the time of enrollment, they had (1) a history of lumbar spine decompression/laminectomy or fusion surgery in past 6 months, (2) a possible non-musculoskeletal cause for LBP symptoms diagnosis (primary or secondary) at baseline visit (e.g., kidney stones, urinary tract infection), (3) a “red flag” LBP diagnosis in the previous 6 months fracture (e.g., cauda equina syndrome, osteomyelitis, or spinal neoplasm), (4) a neurological disorder resulting in moderate to severe movement dysfunction, or (5) the presence of any psychotic disorder.

### Study interventions and participant assignment

Participants were recruited sequentially from potentially eligible patients meeting all eligibility criteria and providing informed consent. When 4–6 participants had been successfully enrolled, they were assigned to a group and that group was randomly assigned to one of 3 study groups: control (no SMP and no HBCC), SMP only (no HBCC), and SMP + HBCC. Groups were assigned in a 2:1:3 allocation to control, SMP only, or SMP + HBCC.

There were two experimental interventions under investigation in this pilot study (Table 1). The first was an evidence-based 6-week SMP led by a registered nurse. The SMP was based on the Arthritis Self-Management Program and the Chronic Disease Self-Management Program [36–39]). Each weekly session was scheduled to last approximately 60 min. The second was a telephone-based HBCC intervention [26, 27, 40] using principles and practices of motivational interviewing [41–43] to increase patient activation and self-management behavior, reduce pain and disability, and improve quality of life. HBCC was delivered in a series of 3 telephone calls: 1 before the SMP started and 2 during the SMP. The first call lasted approximately 30 min and focused on identifying patient expectations for the program, identifying goals and concerns related to cLBP, and using 10-point scales to establish the importance of taking an active role in self-management and the patient’s confidence regarding participation in the SMP sessions. In administering the scales, the nurse interventionist used open-ended questions, affirmations, reflection, and summarization (OARS) methodology [41] to develop a behavior change plan with the participant. The second and third calls were up to 30 min in duration and served as booster sessions in which the nurse interventionist revisited the patient’s goals and barriers and the behavior change plan in relation to the SMP sessions.

**Table 1:** Self-management program sessions and health behavior change counseling telephone calls in pilot study of adults with chronic lower back pain from July 2022 through April 2023

Program by Session No.	Topics	Content and Patient Activities
**SMP**		
1	Overview of self-management of chronic back pain	Acute vs. chronic illness; self-management principals; problem-solving, action planning, and finding resources; symptom management; assign homework
2	Mind-body connection	Relaxation techniques, distraction, positive thinking and self-talk, imagery, and prayer or spirituality; exercise and physical activity; review and assign homework
3	Communication with family, friends, and health care professionals	Communication goals; expressing feelings with “I” messages, minimizing conflict, asking for help, listening, and body language; P.A.R.T. (Prepare, Ask, Repeat, and Take action); mid-program recap and check-in; review and assign homework
4	Healthy eating and weight management	Food choices and flexibility; nutrients, inflammation and inflammatory foods, and vitamins and minerals; eating your thoughts; healthy weight management; review and assign homework
5	Managing medications and making treatment decisions	Mind power and expectations; taking multiple medications; reading a prescription label; taking medicine; questions to ask self about treatment decisions; review and assign homework
6	Planning for the future	Physical concerns about day-to-day living; finding help; looking back and planning for the future; program summary and check-in
**HBCC**		
1	Health behavior change plan	Discuss expectations for SMP; explore goals and identify concerns related to chronic low back pain; use importance and confidence scales to explore beliefs and ability to participate; use OARS to develop health behavior change plan
2	Booster 1	Reflect on goals and barriers in relation to the health behavior change plan and the SMP sessions
3	Booster 2	Reflect on goals and barriers in relation to the health behavior change plan and the SMP sessions

HBCC, health behavior change counseling; OARS, open-ended question, affirmation, reflection, summarization; SMP, self-management program

#### Study outcomes

The study was conducted to assess the potential for a larger clinical trial to determine the effectiveness of augmenting an evidence-based 6-week SMP led by a registered nurse with a telephone-based HBCC intervention.

### Study acceptability and feasibility

We defined *acceptability* as having at least 50% of individuals approached agree to eligibility screening for the study, having at least 30% of those who were deemed eligible for the study agree to participate (i.e., to become “enrolled participants”), and having the enrolled population be representative of individuals residing in the Baltimore metropolitan region. Our threshold of 30% of eligible individuals to become enrolled participants was based on a review of the literature of feasibility for similar interventions with feasibility thresholds ranging from 20 to 50% of eligible participants and stakeholder input that included individuals with chronic low back pain, healthcare providers, and members of the research team. We defined *feasibility* using 2 broad measures: (1) having at least 80% of enrolled participants attend at least 3 of the 6 scheduled self-management program sessions; and (2) having a loss to follow-up rate of < 20% of enrolled participants over the 26-week study.

### Patient activation

Patient activation, a secondary outcome in our pilot study but the primary outcome for the tested intervention, was assessed using the 13-item Patient Activation Measure (PAM) [44]. For each of the 13 items on the PAM, patients were provided 5 response options, ranging from “strongly agree” to “strongly disagree.” Based on their answers, patients were assigned a numerical score ranging from 0 to 100, and the score was used to stratify patients into 1 of 4 stages of activation: stage 1 (believes taking an active role is important), stage 2 (has the confidence and knowledge to take action), stage 3 (takes action), and stage 4 (stays the course under stress) [44]. The PAM has been shown to be a reliable and valid instrument to assess patient engagement in multiple patient populations, including older individuals [45] and those with multiple sclerosis [46] or spine-related pathology [47].

#### Pain-related disability

Secondary outcomes were pain-related disability assessed using the Oswestry Disability Index (ODI) and selected domains from the Patient Reported Outcome Measurement Information System 29-Item (PROMIS-29) Health Profile, version 2.0. Outcomes were assessed at baseline, at 12 weeks, and at 6 months. Pain-related disability was assessed using the ODI, a 10-item measure of low back pain–related disability that evaluates the current effect of a patient’s low back pain on various aspects of daily living. ODI scores range from 0 to 100, with higher scores indicating greater disability [48–50]. The ODI is a reliable and valid assessment of pain-related disability in this population [51].

The PROMIS-29 version 2.0 Health Profile assesses pain intensity using a single 11-item numeric rating scale, 7 health domains (physical function, fatigue, anxiety, depression, sleep disturbance, ability to participate in social roles and activities [i.e., social roles], and pain interference), and a 6-point Likert scale (e.g., “never,” “rarely,” “sometimes,” “often,” “always,” and “not at all”) [52]. The response timeframe was the past 7 days. The score for each health domain was reported on a T-score metric (mean, 50; SD, 10 points) centered on the mean of a sample that matched the 2000 U.S. Census with respect to age, sex, race, and education [53]. For the purposes of our analysis, we focused on physical function, social roles, and pain interference. PROMIS-29 has been demonstrated to be a reliable and valid assessment of health in the general adult population and among patients with chronic pain conditions [54, 55].

### Covariates and statistical analysis

Self-reported social determinants of health were age, gender, educational attainment, and household income. Educational attainment was stratified as < 4-year degree, 4-year degree, and > 4-year degree. Household income was stratified as < $30,000, $30,000–$80,000, and > $80,000 per year.

The presence of comorbid health conditions was assessed using the Charlson Comorbidity Index [56]. Patients reported whether a doctor or health care provider had ever told them that they had any of the following: myocardial infarction, congestive heart failure, peripheral vascular disease, cerebrovascular disease (except hemiplegia), dementia, chronic pulmonary disease, arthritis or other connective tissue diseases, ulcer disease, mild liver disease, diabetes (without complications), diabetes with end organ damage, hemiplegia, moderate or severe renal disease, solid tumor (non-metastatic), leukemia, lymphoma or multiple myeloma, moderate or severe liver disease, metastatic solid tumor, or AIDS. We used the Elixhauser scoring algorithm to estimate 10-year survival [57].

We estimated observed differences in health outcomes across the study groups using mean and standard deviation of the change from baseline at weeks 12 and 26 to estimate effect size and used Hedges’ statistic to account for unequal sample size in the study groups [58].

All statistical analyses were conducted using Stata BE, version 17 (Stata Corp, College Station, TX).

## Results

### Study acceptability and feasibility

**Fig. 1:**
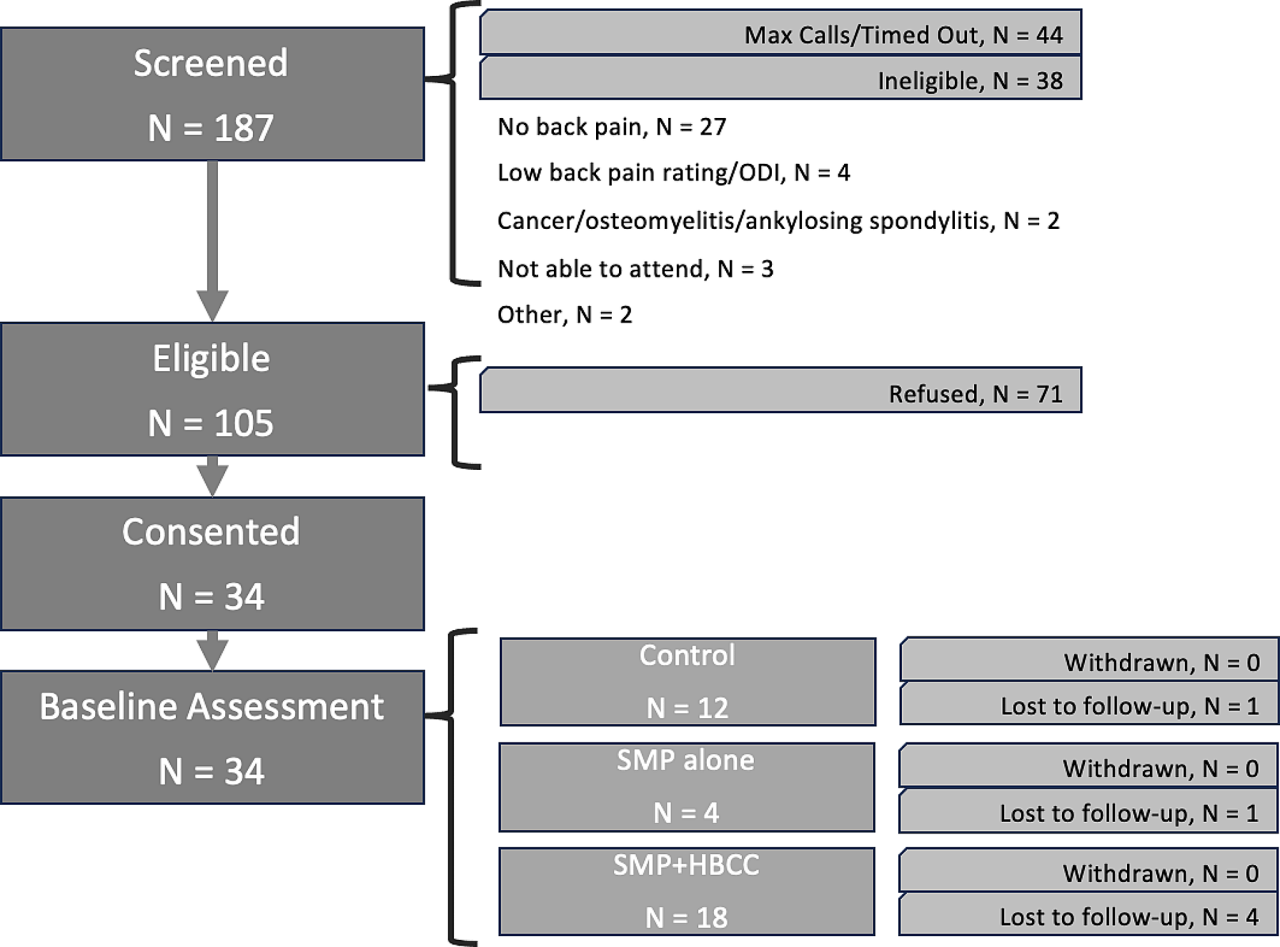
Recruitment flow-chart for a pilot study of adults with chronic lower back pain, July 2022 through April 2023

We identified 187 potentially eligible individuals for screening, of whom we were unable to contact 44 (24%). Of the 143 participants who were screened, 105 (73%) were found to be eligible. Of those who were eligible, 71 (68%) refused and 34 (32%) accepted participation in our pilot study (Fig. 1). Among those who refused participation, the most common reasons were “not interested,” reported by 35 individuals, and “too much time required,” reported by 20 individuals.

The enrolled population included 19 (56%) women, 22 persons who were married or living with a significant other (65%), 13 Black or African American persons (38%), and 4 Hispanic or Latino persons (12%). The mean (standard deviation, SD) age was 45 (10) years of age, and the mean (SD) body-mass index (BMI) was 29 (8.1) kg/m^2^ (Table 2). These individuals were representative of the demographic and clinical characteristics of the Baltimore metropolitan region [59]. Thus, according to our a priori threshold for recruitment, our pilot study demonstrated the acceptability of a larger clinical trial.

**Table 2:** Sociodemographic characteristics of enrolled participants in a pilot study of adults with chronic lower back pain at baseline study visit, overall and stratified by self-management program participation, July 2022 through April 2023

Characteristic	*N* (%)			
	Overall (*N* = 34)	No SMP (*n* = 12)	SMP (*n* = 4)	**SMP** + HBCC (*n* = 18)
Age, y	45 ± 10^*^	44 ± 7.8^*^	41 ± 4.6^*^	47 ± 12.5^*^
Female-identifying	19 (56)	4 (33)	3 (75)	12 (67)
Body mass index value	29 ± 8.1^*^	29 ± 12^*^	30 ± 5.4^*^	29.5 ± 6.0^*^
Marital status				
Single/widowed/divorced	11 (32)	5 (42)	1 (25)	5 (28)
Living with partner	2 (5.9)	0 (0)	1 (25)	1 (5.6)
Married	20 (59)	6 (50)	2 (50)	12 (67)
Refused	1 (2.9)	1 (8.3)	0 (0)	0 (0)
Race^†^				
American Indian/Native American	2 (5.9)	1 (8.3)	1 (25)	0 (0)
Asian	1 (2.9)	1 (8.3)	0 (0)	0 (0)
Black/African American	13 (38)	4 (33)	1 (25)	8 (44)
Native Hawaiian/Pacific Islander	0 (0)	0 (0)	0 (0)	0 (0)
White	19 (56)	8 (67)	2 (50)	9 (50)
Other	2 (5.9)	0 (0)	1 (25)	1 (5.6)
Hispanic	4 (12)	1 (8.3)	2 (50)	1 (5.6)
Education				
Less than high school	1 (2.9)	0 (0)	0 (0)	1 (5.6)
High school	14 (41)	8 (67)	1 (25)	5 (28)
College or more	18 (53)	3 (25)	3 (75)	12 (67)
Refused	1 (2.9)	1 (8.3)	0 (0)	0 (0)
Work status				
Not employed outside of home	5 (15)	2 (17)	1 (25)	2 (11)
Employed part-time	4 (12)	1 (8.3)	1 (25)	2 (11)
Employed full-time	17 (50)	6 (50)	2 (50)	9 (50)
Not employed because of back	7 (21)	2 (17)	0 (0)	5 (28)
Retired	1 (2.9)	1 (8.3)	0 (0)	0 (0)
Household income category, USD				
<5,000	3 (8.8)	3 (25)	0 (0)	0 (0)
5,000–$9,999	1 (2.9)	0 (0)	1 (25)	0 (0)
10,000–29,999	5 (15)	2 (17)	0 (0)	3 (17)
30,000–49,999	3 (8.8)	1 (8.3)	0 (0)	2 (11)
50,000–79,999	6 (18)	1 (8.3)	3 (75)	2 (11)
*≥*80,000	11 (32)	1 (8.3)	0 (0)	10 (56)
Refused	5 (15)	4 (33)	0 (0)	1 (5.6)
Comorbid conditions^‡^				
Anxiety	16 (47)	5 (42)	2 (50)	9 (50)
Cancer	3 (8.8)	0 (0)	0 (0)	3 (17)
Depression	17 (50)	7 (58)	2 (50)	8 (44)
Diabetes	2 (5.9)	2 (17)	0 (0)	0 (0)
Osteoporosis	0 (0)	0 (0)	0 (0)	1 (5.6)
Substance use disorder	1 (2.9)	1 (8.3)	0 (0)	1 (5.6)
Health care utilization				
Computed tomography	7 (21)	2 (17)	2 (50)	3 (17)
Electromyelography/nerve study	7 (21)	2 (17)	3 (75)	2 (11)
Epidural steroid injections	9 (27)	1 (8.3)	2 (50)	6 (33)
Magnetic resonance imaging	18 (53)	6 (50)	3 (75)	9 (50)
Radiography	18 (53)	5 (42)	3 (75)	10 (56)
Surgery, decompression	6 (18)	2 (17)	1 (25)	3 (17)
Surgery, fusion	2 (5.9)	1 (8.3)	0 (0)	1 (5.6)
Other	8 (24)	3 (25)	2 (50)	3 (17)
Opioid use in last 3 months				
Daily/almost daily	4 (12)	1 (8.3)	0 (0)	3 (17)
A few times	7 (21)	0 (0)	4 (100)	3 (17)
None	23 (68)	11 (92)	0 (0)	12 (66)

SMP, self-management program

^*^Expressed as mean ± standard deviation

^†^Participants may identify as more than one race

^‡^Participants who report having this condition currently or in the past

**Fig. 2:**
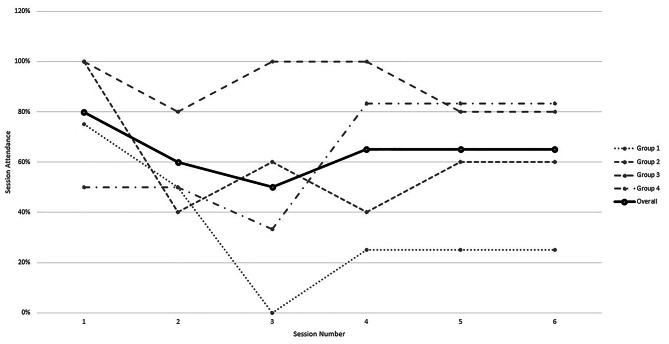
Attendance in the self-management programs in a pilot study of adults with chronic lower back pain, overall and stratified by study group, July 2022 through April 2023

Enrolled individuals participated in 1 of 3 study interventions: control (*n* = 12), SMP alone (*n* = 4), and SMP with HBCC (*n* = 18). For those taking part in the web-based SMPs (SMP alone and SMP with HBCC), there were 4 separate groups that met once a week for 6 weeks. Among the 22 individuals in these groups, 20 (91%) participants attended at least 1 group session, 15 (68%) attended at least 3 sessions, and 7 (32%) attended all 6 sessions. Six participants (18%) were lost to follow-up over the course of the 26-week study. There were no baseline differences between participants who were or were not lost to follow-up with respect to age, gender, BMI, pain-related disability, or worst back pain intensity. Attendance in the self-management sessions increased over the course of the study (Fig. 2). In the first two groups, 4 of 9 participants (44%) attended at least 3 sessions. In the second two groups, 11 of 13 participants (85%) attended at least 3 sessions. Based on the increased attendance over the course of the study, the pilot study met 1 of the 2 thresholds for feasibility (“loss to follow-up rate ≤ 20), providing evidence for the feasibility of a larger clinical trial.

### Patient activation

The mean (SD) patient activation score at baseline for enrolled participants was 57 (16.5) (Table 3). There were 12 participants in stage 1, 4 in stage 2, 6 in stage 3, and 12 in stage 4. There were no differences in patient activation scores between those receiving no SMP (51 (20)), those receiving SMP (54 (18)), and those receiving SMP + HBCC (62 (13)).

**Table 3:** Patient-reported outcome measures in a pilot study of adults with chronic lower back pain, July 2022 through April 2023

Characteristic	Mean ± Standard Deviation			
	Overall (*N* = 34)	Control (*n* = 12)	SMP (*n* = 4)	**SMP** + HBCC (*n* = 18)
Back pain intensity				
Current	4.9 ± 2.2	5.6 ± 1.2	2.8 ± 2.1	5.0 ± 2.5
Worst in past 24 h	7.3 ± 1.6	7.2 ± 1.9	7.8 ± 0.5	7.3 ± 1.6
ODI value	42 ± 12	41 ± 8.8	42 ± 14	42 ± 13
Patient activation score	57 ± 16.5	51 ± 20	54 ± 17.5	62 ± 13
Patient activation stage				
1	12 (35)^*^	7 (58)^*^	2 (50)^*^	3 (17)^*^
2	4 (12)^*^	0 (0)^*^	0 (0)^*^	4 (22)^*^
3	6 (18)^*^	1 (8)^*^	1 (25)^*^	4 (22)^*^
4	12 (35)^*^	4 (33)^*^	1 (25)^*^	7 (39)^*^
PSOCQ				
Pre-contemplative	2.9 ± 0.5	2.9 ± 0.4	3.1 ± 0.5	2.8 ± 0.5
Contemplative	3.9 ± 0.6	3.9 ± 1.0	3.8 ± 0.4	3.9 ± 0.5
Action	3.3 ± 0.8	3.6 ± 0.8	3.1 ± 0.8	3.3 ± 0.8
Maintenance	3.6 ± 0.8	3.8 ± 0.8	3.4 ± 0.6	3.6 ± 0.8
PROMIS-29 profile				
Anxiety	56 ± 8.4	54 ± 5.4	61 ± 9.0	57 ± 9.7
Depression	55 ± 8.6	54 ± 6.3	56 ± 14	55 ± 9.2
Fatigue	56 ± 11	53 ± 8.2	53 ± 9.2	58 ± 14
Pain interference	61 ± 6.7	57 ± 6.0	63 ± 9.7	63 ± 5.5
Physical function	40 ± 6.6	40 ± 6.2	43 ± 4.1	39 ± 7.3
Sleep disturbance	55 ± 7.1	53 ± 7.1	56 ± 8.5	57 ± 7.2
Social role participation	45 ± 10	43 ± 7.1	41 ± 7.8	47 ± 12

ODI, Oswestry Disability Index; PROMIS, Patient-Reported Outcomes Measurement Information System; PSOCQ, Pain Stages of Change Questionnaire

^*^Expressed as n (%)

Participants in the SMP + HBCC endorsed an approximately 4-point improvement in patient activation at the 12- and 26-week assessments (Table 4). There were no observed improvements in patient activation in the control and SMP groups at these two time points. Improvements in patient activation for participants in the SMP + HBCC were of medium-to-large effect size compared to those in the control group and of medium effect size compared to those in the SMP group (effect size at 12 weeks: SMP + HBCC vs. control, 0.39 and SMP + HBCC vs. SMP, 0.22; effect size at 26 weeks: SMP + HBCC vs. control, 0.68; SMP + HBCC vs. SMP, 0.23).

**Table 4:** Change from baseline at 12- and 26-weeks in select patient-reported outcome measures in a pilot study of adults with chronic lower back pain, overall and stratified by study group, July 2022 through April 2023

Characteristic	Change from Baseline (Mean ± SD)				Hedges’ Effect Size		
	Overall	Control	SMP	SMP HBCC	SMP Alone vs. Control	SMP + HBCC vs. Control	SMP Alone vs. SMP + HBCC
Patient activation score							
12 weeks	2.3 ± 9.2	0.2 ± 8.8	1.7 ± 9.7	3.9 ± 9.5	0.16	0.39	0.22
26 weeks	1.6 ± 8.3	-1.7 ± 5.1	1.6 ± 10.9	3.8 ± 9.2	0.46	0.68	0.23
ODI value							
12 weeks	–3.6 ± 8.3	–0.6 ± 10	–2.7 ± 4.2	–5.9 ± 7.3	–0.22	–0.62	–0.46
26 weeks	–3.6 ± 9.9	–2.3 ± 13	–2.0 ± 11	–5.1 ± 7.1	–0.42	–0.90	–0.40
PROMIS-29 profile							
Physical function							
12 weeks	4.6 ± 6.3	3.6 ± 4.4	5.8 ± 6.5	5.1 ± 7.6	0.45	0.23	–0.09
26 weeks	4.8 ± 8.3	1.8 ± 3.7	2.6 ± 8.4	6.3 ± 11	–0.24	0.28	0.34
Social role participation							
12 weeks	4.8 ± 8.2	2.6 ± 4.0	4.9 ± 6.8	6.2 ± 11	0.62	0.42	0.13
26 weeks	4.7 ± 8.4	1.4 ± 4.8	2.8 ± 5.6	6.4 ± 11	–0.12	0.33	0.34
Pain interference							
12 weeks	–2.1 ± 8.2	3.5 ± 4.0	–3.8 ± 2.6	–5.5 ± 9.1	–1.90	–1.20	–0.21
26 weeks	–4.1 ± 5.0	1.1 ± 4.2	–2.9 ± 8.9	–6.1 ± 4.2	–0.15	–0.95	–0.62

HBCC, health behavior change counseling; ODI, Oswestry Disability Index; PROMIS, Patient-Reported Outcomes Measurement Information System; SD, standard deviation; SMP, self-management program

### Pain-related disability

Mean (SD) worst back pain for enrolled participants was 7.3 (1.6), and pain-related disability on the Oswestry Disability Index was moderate (42 ± 12) (Table 3). Participants endorsed moderate impairments in physical function (40 ± 6.6) and social roles (45 ± 10) and moderately severe pain interference (61 ± 6.7).

Participants in the SMP and SMP + HBCC reported improvements in physical function and social roles and reduction in pain-related disability and pain interference at the 12- and 26-week assessments (Table 4). The control group did not show any appreciable changes in these measures over the course of the study. Compared to the control group, participants in both the SMP and the SMP + HBCC groups demonstrated improvement in health outcomes, with small-to-medium effect size differences in pain-related disability and physical function and large effect size differences in social role participation and pain interference at the 12-week assessment. These improvements persisted in the SMP + HBCC group at the 26-week assessment, with small-to-medium effect size differences in physical function and social role participation and large effect size differences in pain-related disability and pain interference. Comparing the two SMP groups, participants who received the HBCC augmentation demonstrated medium effect size differences in pain-related disability at both time points and medium effect size differences in physical function, social role participation, and pain interference at the 26-week assessment.

## Discussion

Our pilot study of a 6-week, evidence-based SMP delivered using a synchronous, video-based web platform with or without augmentation using telephone-based HBCC demonstrated the feasibility and acceptability of the proposed clinical trial, allowed us to refine the intervention materials, and provided observed effect size differences that can guide estimates of statistical power and sample size for a larger clinical trial.

The enrolled population in our study was similar to the populations of other self-management studies for those living with cLBP in terms of both demographic and clinical characteristics [60–63]. Where our study differed was in the inclusion of a racially diverse population. Roughly 40% of our study population identified as Black or African American, which is similar to the demographic characteristics of the Baltimore metropolitan region [59]. The inclusion of underrepresented individuals in our study improved the generalizability of the study.

Among the 22 participants assigned to an SMP group (with or without HBCC), 15 attended at least 3 sessions of their assigned. This was considered a minimal dose of the self-management program. Attendance improved over the course of the study and was affected by participants’ knowledge of the program and their personal drive to take part.

Participants in the SMP + HBCC group endorsed improvements in patient activation compared to the control and SMP groups that were of a magnitude similar to other reported interventions to improve patient activation [26, 27]. Those in the SMP and SMP + HBCC groups reported improvement in physical function and social roles and reduction in pain-related disability and pain interference following group participation. Effects were larger in the group that received HBCC augmentation. The improvements in health outcomes observed at the 26-week assessment potentially demonstrate that participants in these groups were able to manage cLBP flares with the skills acquired. This provides support for a larger clinical trial that can rigorously test the effectiveness of SMP + HBCC in patients with cLBP.

Our study was not without limitations. It was our original intention to sequentially enroll groups of 4–6 participants in each group to be assigned to control, SMP, or SMP + HBCC in a 2:1:3 fashion because our main interest was in the comparison between control and SMP + HBCC. With loss to follow-up, this resulted in only 4 participants receiving the SMP. This small sample size may have made our effect size estimates susceptible to outliers.

### Challenges and lessons learned

In this single-masked pilot study of a web-based SMP with or without HBCC augmentation, we successfully delivered 6 weekly sessions on pain self-management strategies such as positive thinking and self-talk, expressing feelings with “I” statements, and asking questions about treatment decisions. We experienced several challenges during the study that, upon resolution, improved our performance on several key metrics.

### Study versus patient goals

Our outcomes measures, while important, did not always align with the goals that individuals had for managing their back pain. Many participants voiced strong desires to resume physical activities they previously enjoyed, such as being physically active with their children or grandchildren, gardening, hiking, running, and taking long car rides and flights. Several participants also expressed a desire to sleep for more than a few hours at a time. Barriers to achieving these goals included debilitating pain and an inability to sit, stand, or lay down for more than a few minutes to a few hours at a time due to the resulting pain. While our outcome measures focused on pain intensity and its interference with activities such as standing or sitting, we did not specifically focus on higher-order social goals.

### Attendance, duration, and community

Attendance in the sessions was reportedly affected by the participants’ knowledge of the program as well as their personal drive to attend. Participants in the first 2 SMP groups were often difficult to reach by telephone. After a few sessions, participants found benefits they had not expected at the start of the program. Most of them mentioned a sense of community and understanding with other participants as a benefit of their participation. Participants said that knowing there were other people who faced similar problems made self-management easier. They reported a feeling of “relief” and “comfort” in knowing they were not alone in their daily struggle with chronic back pain.

Several participants mentioned that they had not met or talked with other people with cLBP before the SMP sessions. A majority of participants said their favorite aspect of the SMP sessions was the sense of community they felt in talking with others who shared their experience. The groups with the highest level of engagement (groups 3 and 4) said they were sad that the SMP sessions were ending and voiced a strong desire for an 8-to-10-week program. Several participants reported that the group members were just starting to feel comfortable with each other when the SMP ended.

### Assumptions about content

We made some assumptions about the participants’ general knowledge of self-management programs. For example, we assumed that the term “self-management program” was a self-evident term; however, many participants reported a lack of knowledge about what self-management entailed. It was only through participation in the program that participants gained a better understanding of the practices and tools they could use to manage their back pain flares. Of the SMP sessions, the one about building effective communication strategies with health care providers, family, and friends was most often cited as providing new information and as the most beneficial session. Participants also mentioned the resounding benefit of the session on managing medication and making treatment decisions. Finally, participants appreciated the inclusion of community-based resources for those living with cLBP.

### Session timing, format, and platform

Participants’ opinions about the optimal time of day to conduct the sessions were mixed. Some felt that the weekday evening time slot was best because most people are available after typical workday hours. However, participants with small children expressed a desire for sessions that were not during dinner hours (e.g., 5–7 p.m. or 8–10 p.m.). Other participants suggested conducting some or all sessions during the weekend to better accommodate participants’ work schedules.

While the web-based format was found to be acceptable, several participants suggested conducting at least 1 in-person session to allow participants to get to know each other and a longer in-person session at the end to reflect on the sessions and conclude the program. There was agreement among some that in-person SMP sessions would enhance the group’s camaraderie and expedite the experience of sharing personal details with other group members. One participant explained that a virtual platform presented more distractions—for example, with kids and pets in the background—and made it difficult for participants to build rapport.

## Conclusions

This single-masked randomized pilot study demonstrated the acceptability and feasibility of delivering a web-based SMP augmented with HBCC for those living with cLBP and was associated with improvements in health outcomes that persisted up to 26 weeks. Important lessons were learned regarding content, attendance, content, and platform, and they can be used to inform the development of these interventions in the future. Additional research is needed to develop robust evidence for the adoption of SMP + HBCC to support patients managing their cLBP.

### Electronic supplementary material

Below is the link to the electronic supplementary material.

Supplementary Material 1

Supplementary Material 2

## Data Availability

The datasets generated and/or analyzed during the current study are not publicly available due restrictions on data sharing by our institutional review board but are available from the corresponding author on reasonable request.

## Abbreviations

BMI: body mass index
cLBP: chronic low back pain
HBCC: health behavior change counseling
LBP: low back pain
OARS: open-ended question, affirmation, reflection, summarization
ODI: Oswestry Disability Index
PROMIS: Patient-Reported Outcomes Measurement Information System
SD: standard deviation
SMP: self-management program
SMP + HBCC: self-management program augmented with health behavior change counseling
