# Supplementary figures and images for: Nurse-led web-based self-management program to improve patient activation and health outcomes in patients with chronic low back pain: an acceptability and feasibility pilot study
Source: BMC Nurs. 2024 Jul 31;23:524. doi: 10.1186/s12912-024-02155-w (PMC11293200; doi:10.1186/s12912-024-02155-w)

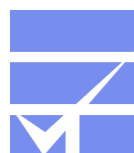

# CONSORT

TRANSPARENT REPORTING of TRIALS

## CONSORT 2010 Flow Diagram

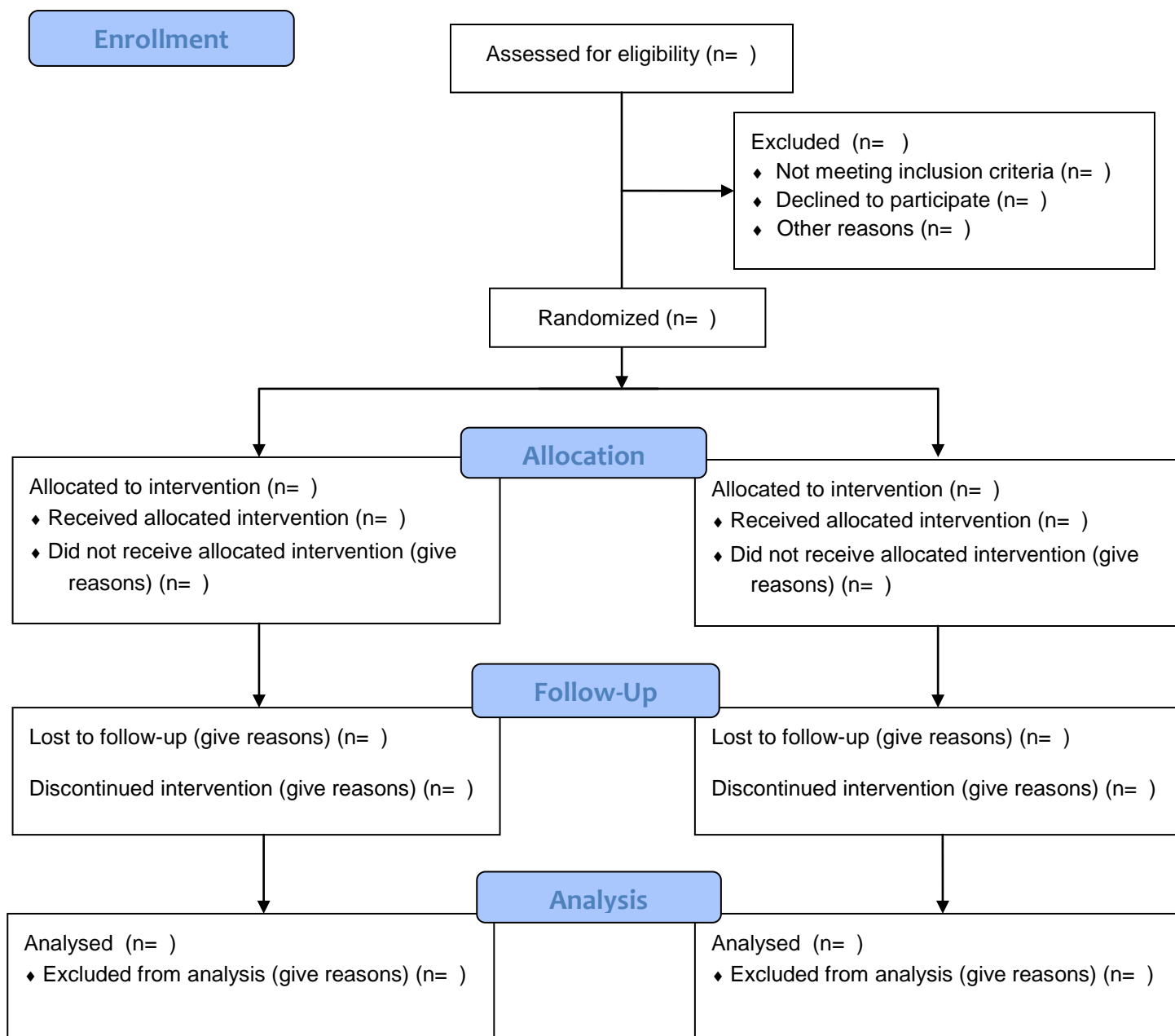

Supplement: Supplementary file 1 — Supplementary Material 1 [file 12912_2024_2155_MOESM1_ESM.pdf]
